# Resveratrol for Weight Loss in Obesity: An Assessment of Randomized Control Trial Designs in ClinicalTrials.gov

**DOI:** 10.3390/nu14071424

**Published:** 2022-03-29

**Authors:** Ashley Hillsley, Vanessa Chin, Amy Li, Craig S. McLachlan

**Affiliations:** 1 Centre for Healthy Futures, Torrens University Australia, Pyrmont, NSW 2009, Australia; ahillsley@torrens.edu.au (A.H.); vanessa.chin@torrens.edu.au (V.C.); craig.mclachlan@torrens.edu.au (C.S.M.); 2 Department of Pharmacy & Biomedical Sciences, La Trobe University, Bendigo, VIC 3550, Australia

**Keywords:** resveratrol, obesity, weight loss, randomized controlled trial, clinical trial design

## Abstract

Resveratrol is a polyphenol that may improve weight loss outcomes in obese individuals. However, assessing the effectiveness of resveratrol supplementations as an appropriate intervention for weight loss in obesity across randomized control trials (RCTs) has been complicated by variability in their design. This study aims to evaluate design elements across RCTs of resveratrol interventions in obesity with weight loss as an end-point outcome, as recorded in ClinicalTrials.gov. We found discrepancies in participant inclusion criteria (sample size, age ranges, sex, BMI, medical conditions), interventional design (delivery modalities, dosages, duration) and primary outcomes measured (anthropomorphic, blood biomarkers). We identified a near three-fold variation in study sample size, two-fold variation in minimum inclusion age, five modalities of therapeutic resveratrol delivery with interventional durations ranging from two weeks to six months. Weight loss was only identified as a primary outcome in three of the seven studies evaluated. In conclusion, heterogeneity in trial design using resveratrol suggests that weight-loss-related outcomes are difficult to interpret and cross-validate. Indeed, conclusions drawn from human studies have been inconsistent, which may be attributed to study design heterogeneity including major differences in sample population, age, sex, BMI, underlying health conditions and end-point measures.

## 1. Introduction

Resveratrol is a natural polyphenol compound found abundantly in select plants such as the skin of red wine grapes, peanuts, and blueberries [1]. This polyphenolic compound has been reported to have anti-obesity and metabolic reprogramming properties in translational models [2,3]. In animal studies, resveratrol was beneficial in reducing obesity-related adverse metabolic outcomes including insulin resistance, body weight, adipose tissue weight and size [4,5,6]. In comparison, evidence indicative of the positive effects of resveratrol on obesity outcomes in humans have led to inconsistent findings. A handful of clinical trials examined the impact of resveratrol on human obesity; however, their findings were heterogenous with some studies showing a positive reduction in body weight [7,8,9,10,11] while others demonstrate no influence on body weight but did observe other metabolic benefits [12,13,14,15,16,17,18,19].

Despite the significant progress made in determining the effects of resveratrol in translational studies with animal models of obesity, the heterogeneity in design elements in human randomized control trials (RCT) and meta-analysis inclusion criteria pose a significant challenge in drawing evidenced-based conclusions. For example, Delpino et al. (2021) conducted a systematic review of resveratrol supplementation that was found not to have anti-obesity effects as measured by body weight, BMI or waist circumference [15]. Nonetheless, the authors also noted that three of the nineteen RCTs included in the analysis demonstrated positive outcomes on obesity but cautioned that these benefits may be attributed to co-morbidities (e.g., diabetes, metabolic syndrome) or co-interventions (i.e., orlistat). Additionally, Batista-Jorge et al. (2020) performed a randomized control study that suggested lifestyle modification associated with resveratrol produced improvements in several parameters including body weight, BMI and waist circumference [12]. While there was a significant reduction in anthropometric parameters within the resveratrol group before and after the prescribed lifestyle intervention (physical activity and nutrition), there was no difference between the resveratrol and placebo groups. Therefore, the lack of standardization in study design and assessment of measured outcomes specific to resveratrol, or with the combination of other therapeutic compounds of synergic interest, would dilute the ability to evaluate compound efficacy in pooled data studies. Indeed, this has been the case in systematic reviews and meta-analysis, although it has not been formally assessed.

Obesity is a multi-systemic and multi-factorial condition with initial presentations of high body weight, BMI and waist circumference along with complex co-morbidities of high blood pressure, insulin resistance, diabetes, cardiovascular disease, metabolic syndrome, fatty liver disease, and cancers [20]. Given the complexity of obesity, clear standardization in study design, inclusion and exclusion criteria, primary and secondary outcome measures and in primary and secondary end points are necessary across reportable RCT’s. Such variables should ideally include the dosage and frequency of resveratrol administration, the route of administration, duration of intervention, single or multi-compound intervention, types of weight loss measures, interval of measures, co-morbidities, and population demographics and whether this heterogeneous or homogenous with respect to health, age, BMI, sex and population sample size.

The World Health Organization (WHO) has defined an interventional clinical trial as one that assigns humans to one or more interventions that evaluates health outcomes of the intervention. All trials must be registered before participant recruitment can commence in line with recommendations made by the WHO, the International Committee of Medical Journal Editors and regulatory authorities such as the FDA. The largest and most extensive trial registry for RCTs is ClinialTrials.gov, which we used for our review of RCT trials using resveratrol. This repository lists a number of studies conducted in 220 countries and includes the following subsections: study description, study design, details of study arms and interventions, outcome measured (primary and secondary), eligibility criteria, contacts and locations and further information. Using ClinicalTrials.gov, we aimed to interrogate the inclusivity of published trials, whether they met our definition of inclusion/exclusion criteria and to identify discrepancies in the design of resveratrol interventions on obesity using weight loss as an end-point measure. It is not within the aim of this study to systematically evaluate the effectiveness of resveratrol.

## 2. Materials and Methods

### 2.1. RCT Identification, Screening, and Eligibility

**Figure 1:**
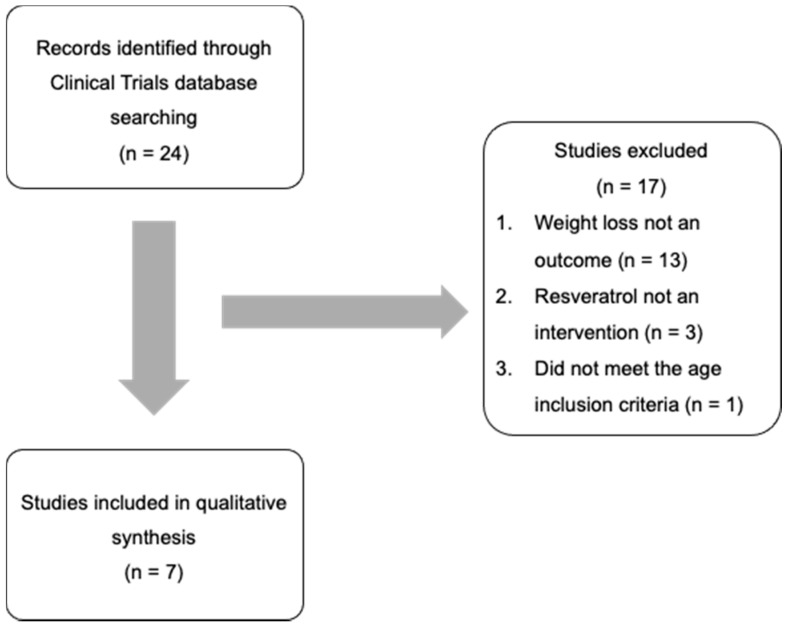
Flowchart of clinical trial selection process for inclusion into this study.

The U.S. Library of Medicine registry for clinical trials (https://www.ClinicalTrials.gov/, accessed on 22 February 2022) was used to identify RCT for obesity with a single resveratrol intervention. A search was conducted on 14th of October 2020 based on the search terms ‘Obesity and resveratrol’ or ‘Body weight and resveratrol’ or ‘Pre-Diabetes and resveratrol’, with inclusion parameters ‘completed trials’, and ‘Adult (18–64)’. This search strategy resulted in a pool of 24 registered RCT trials (Figure 1). These initial 24 trials were then evaluated in detail against inclusion and exclusion criteria. The inclusion criteria were based on the presence of weight loss as a measured outcome with at least one arm of the trial including obesity. Weight loss could be either a primary or secondary outcome or simply listed in the trial design. Obesity with or without components that define metabolic syndrome were also included. Exclusion criteria included pregnancy, presence of chronic conditions (other than metabolic syndrome) and/or acute interventions. An acute intervention is defined as the lack of sufficient time to observe weight loss. After applying the inclusion/exclusion criteria, 7/24 registered clinical trials were retained for analysis.

### 2.2. Comparison of Trial Elements

Key elements of the trial designs were assessed from the seven RCTs included in the analysis. The variables for RCT design comparison are timing of weight loss measures, anthropometric measures of weight loss (DXA, Ultrasound, MRI, Weight, BMI, and other physical weight related measurements), assessment of inflammatory or immune biomarkers, resveratrol dosage (active constituent concentration/volume), pharmacological dosing of resveratrol (food, refined or mixed dosage, interval timing of dosage), inclusion and exclusion criteria based on demographic composition, and BMI range. The intent of this comparison is to identify the heterogeneity of the study population and trial design.

### 2.3. Comparison of ClinicalTrials.gov Trial Elements versus Those Included in the Most Recent Published Meta-Analysis

A review of PubMed with the same search terms used in the RCT registry, revealed a recent meta-analysis of resveratrol effects in reducing obesity [11]. This meta-analysis was used to contrast clinical design elements with those published in the trials selected from ClinicalTrials.gov. Additionally, we compared whether the meta-analysis included all the published RCT’s that we had identified (Table S1). Furthermore, we were also interested in whether the selection of papers that were incorporated into the meta-analysis used similar inclusion/exclusion criteria as the meta-analysis itself.

### 2.4. Statistics

The extracted data were then collated, tabulated, and narratively synthesized. Data is summarized as either means, ranges or percentages. This section may be divided by subheadings. It will provide a concise and precise description of the experimental results, their interpretation, as well as the experimental conclusions that can be drawn.

## 3. Results

A total of 24 completed clinical trials with resveratrol were initially identified on the clinical trials registry. Of the 24 trials, 17 were excluded based on weight loss not being an outcome (*n* = 13), resveratrol not being the only intervention (*n* = 3), or because it did not meet our age inclusion criteria (*n* = 1). The remaining clinical trials (*n* = 7) examines resveratrol interventions in individuals over the age of 18 with weight loss as an assessed outcome at the end of the trial (Figure 1).

**Figure 2:**
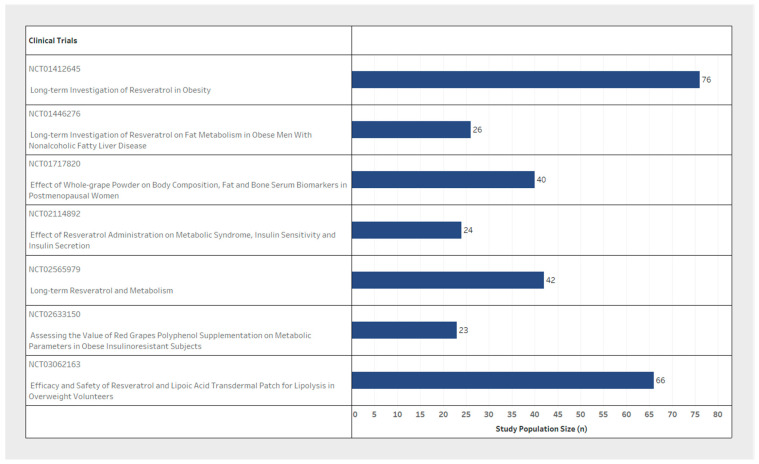
Comparison of participant enrolment size between clinical trials.

**Figure 3:**
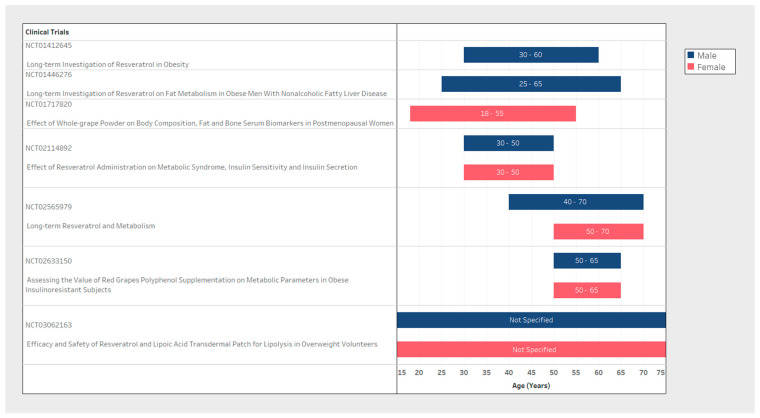
Comparison of participant age range and sex between clinical trials.

Sample size and participant inclusion criteria for each of the seven studies varied significantly between clinical trial design (Table 1). The population sample size ranged from 23 to 76 participants (Figure 2) with considerable variability in basic demographic information. In the sex variable, two studies included only male participants (Figure 3, blue only), while one was a female only sample population exploring post-menopausal effects (Figure 3, red only) and four included both male and female participants (Figure 3, blue and red). Minimum participant age also varied from 18 to 50 years old between trials (Figure 3). Furthermore, despite the design of these studies with the eventual reported outcome of weight loss, two of the seven studies did not explicitly state any BMI, weight, or height limits for participant selection. Only two studies reported an upper BMI limit to their inclusion criteria (Table 1, inclusion criteria).

Interventional design in these clinical trials included resveratrol as a direct oral supplement (*n* = 4), as whole grape powder (*n* = 1), supplementation with grape polyphenols (*n* = 1) and resveratrol delivered by dermal patch (*n* = 1) (Table 1, intervention). The dosage varied by delivery type with oral resveratrol administered in either 75 mg or 500 mg 2–3 times a day, but dosage and frequency were not reported in the grape polyphenol or dermal patch trial. Finally, the total duration of interventional administration ranged from 14 days to 180 days, and further comparison of clinical trials using oral resveratrol revealed study duration ranging from 90 to 180 days.

Primary outcomes and the number of outcomes measured also varied between the included trials (Table 1, outcomes measured). While all trials measured weight loss in some form, only three of the seven clinical trials listed markers of weight loss (e.g., changes to body composition, waist circumference, fat thickness) as a trial primary outcome. Weight loss markers were assessed by a combination of anthropometric (*n* = 3), ultrasound (*n* = 1), magnetic resonance (MR; *n* = 2) and/or dual-energy X-ray absorptiometry (DXA; *n* = 5) at the end of the intervention period. Other biochemical markers such as insulin resistance, glucose tolerance and/or lipid markers were also assessed and acted the primary outcome for five of the seven trials.

Finally, we compared the ClinicalTrials.gov trial elements to those included in the most recently published meta-analysis and found that only nine of the 35 RCTs included in their analysis were registered in ClinicalTrials.gov (Table S1). Of these nine registered RCTs, three overlapped with our search and only one was included based on our specific inclusion criteria (Figure 1). Hence, this RCT included eight trials that we had excluded. Two RCTs we excluded did not meet our criteria with weight loss as a measured outcome (Figure 1, studies excluded). Furthermore, there was an evident lack of consistency in the inclusion criteria of the meta-analysis despite the primary aim of examining weight loss. In 35 studies included in the meta-analysis, overweight or obesity was only evident in 11, diabetes or pre-diabetes was found in nine studies that did not overlap with the overweight-obesity condition, three studies included metabolic syndrome, four included non-alcoholic fatty liver disease, two included hypertension, two assessed cardiovascular disease and four examined healthy individuals (Table S1). All RCTs included in our analysis were overweight or obese as a baseline condition.

## 4. Discussion

In this study, we examined the variability in clinical trial design elements from registered RCTs which utilize resveratrol for obesity-related weight reduction. Our analysis of readily available trial design information deposited in ClinicalTrials.gov clearly suggested differences in population sampling criteria (based upon age, sex, BMI, co-morbidity, and sample size), interventions (dosage and interval), and outcome measures (anthropometric, metabolomic and biochemical markers). Despite our pre-defined screening for normative population data, we could only yield seven studies to include in our analysis.

Our primary finding was that not all registered trials detailed fundamental inclusion and exclusion criteria, which would be necessary for a cross-comparison of baseline and outcome assessments. While trial-to-trial variations are likely associated with variable trial objectives, these studies nonetheless lacked complete trial design information in many cases, making evidence-based cross-comparison problematic with respect to whether resveratrol administration is effective against obesity. This is particularly the case if one tried to pool these seven studies identified from ClinicalTrials.gov to evaluate a mean effect. The small sample size of these studies, ranging from 23 to 76 participants (per study), has the potential to bias findings, given the large biological heterogeneity that exists [21] and that is evident from the high variation of inclusion conditions listed in Table 1 and Table S1. Furthermore, this large degree of heterogeneity inherent to meta-analyses, as exemplified by [11], confounds any interpretations of efficacy in interventional strategies, which is optimized for future clinical applications underlying new trials.

We observed differences for sex allocation across trials where 3/7 studies examined both sexes, whereas 2/7 studies included men exclusively and the remaining 2/7 studies included only women. Sex differences are a well-recognized biological factor in adiposity and obesity-related weight gain common in both humans and rodent models partly attributed to sex-hormone levels that also change with age [22,23,24]. In translational models using mice, a greater decline in inflammation and metabolic parameters was observed in males at a young age but these findings were reversed in females of middle age [25,26]. In a study with post-menopausal women, resveratrol was found to cause no direct changes on serum estrogen or testosterone concentrations but did have positive effects on estrogen metabolism and sex hormone binding globulin that may have a downstream impact on the bioavailability of sex hormones [27]. However, any effects of resveratrol on obesity stratified specifically by sex, age or hormone changes influenced by resveratrol remains to be elucidated. Additionally, it is unlikely that this could be determined in the current trials as the number of participants per trial lacked the statistical power to tease out additional sex or age interactions.

BMI range, a screening tool to identify overweight and obese individuals, was also variable across the seven studies we reviewed. The CDC and WHO classifies adult BMI > 25 kg/m^2^, 30 kg/m^2^ and 40 kg/m^2^ to be overweight, obese and morbidly obese, respectively [28,29]. However, those of Asian ethnicity have lower cut-offs for BMI classification with BMI > 23 kg/m^2^ being classified as overweight and BMI > 27 kg/m^2^ being classified as obese [30]. These published studies identified that individuals of Asian descent with a BMI between 23–24.9 kg/m^2^, although considered within normal range under international guidelines, are at risk of developing obesity-related disorders [31,32]. Our findings show that the seven included studies have a lower-than-expected BMI range from 23 kg/m^2^ to 30 kg/m^2^. These studies do not necessarily account for ethnicity that would physiologically influence the setpoint for weight gain and weight loss—a common measurable outcome across these studies. Thus far, there is limited evidence to suggest associations between ethnicity, differences in pathology between the BMI classification (overweight, obese and morbidly obese), and effects of resveratrol.

Intriguingly, it is not standard practice to utilize BMI to calculate the administration of a daily therapeutic dosage of resveratrol in RCTs. In contrast, animal studies of resveratrol in terms of obesity standardizes the dosage based on mg resveratrol/kg total body weight/day (reviewed in [6]). We found a lack of dosage standardization amongst recruited participants. For example, in an average person of 175 cm, the participant needs to be 70 kg to reach a minimum BMI of 23 identified in our study, 77 kg to achieve a borderline overweight BMI of 25, 92 kg to achieve a borderline obese BMI of 30 and 122 kg to achieve a BMI of 39.9. The weight difference between the minimum (23 kg/m^2^) and maximum BMI (39.9 kg/m^2^) amongst the RCTs included in our study is 52 kg. The average daily dose of resveratrol ranged between 150 mg (low dose) to 1500 mg (high dose). For the low dose category, the standardized daily dose would be 2.1 mg/kg at BMI 23 and 1.2 mg/kg at BMI 39.9. For the high dose category, 21.4 mg/kg at BMI 23 and 12.3 mg/kg at BMI 39.9 is the standardized daily dose. This is significantly lower than the average 30–200 mg resveratrol/kg body weight/day common to animal studies, where significant findings of resveratrol’s effects on obesity have been observed [6]. Perhaps it is possible to extrapolate an optimal human dosage from animal studies [33].

In addition to the need to standardize the therapeutic dosage to body weight or BMI, considerations of total dosage are also necessary where the European Food and Safety Authority in 2016 concluded that 150 mg/day is a safe dosage but noted side effects such as diarrhea and gastrointestinal symptoms in interventional studies administering 1000 mg/day [34], and at 2500 mg/day resulted in nausea, vomiting and liver dysfunction [35]. See [36] for detailed review of health benefits based on resveratrol dosage. These and other side effects occurring at a moderately high dosage (1500 mg/day) may contribute to participant drop off [37] and impact the statistical power of a study [38].

Another essential consideration is the route of delivery, including formulation of oral administration, topical application or injections. It is well established that oral application of resveratrol has a paradoxical relationship between bioavailability and bioactivity whereby the molecule is well absorbed (~75%) by the body but is also rapidly metabolized by the liver resulting in short-lived activity in the circulation, which suggests limited chronic clinical efficacy [39,40]. The topical or trans-dermal delivery of resveratrol has been observed in the treatment of skin conditions improving inflammation, sun damage and ageing characteristics [41] but have not yet yielded peer-reviewed results in the management of obesity or other chronic conditions. The available evidence points towards the ease of application in utilizing oral administration in humans, with an oral or injection route of administration established in mice models of obesity.

Of final consideration is the end-point measures. In our study, we focused on weight loss, as measured by body weight, waist circumference, BMI and body composition as the primary end point for resveratrol efficacy on obesity. There is, however, heterogeneity in the clinical end-point measurements to assess weight changes. Different modalities were used to assess the pooled evidence of resveratrol on weight loss. Of the seven studies assessed, only three studies used anthropometric measurements, one used ultrasound, two used an MRI and all but three examined DXA alone or in combination with other modalities.

Resveratrol has been shown to have a potentially positive effect on body composition parameters in both human and rodents. In humans, resveratrol also improved biochemical markers including liver lipid infiltration [16], triglycerides [7,19], cholesterol [42], blood pressure [42], insulin sensitivity [9,42] and pro-inflammatory markers [8,19]. Intriguingly, as reviewed in [6], the impacts of these improved physical and biochemical biomarkers as a result of resveratrol administration were not observed at extremely low (75 mg/day) or high doses (3000 mg/day) [13,43]. However, not all RCTs included in our study examined biochemical biomarkers and for those that did, we found heterogeneity in which biomarkers were actually assessed.

**Limitations and future considerations.** Firstly, in our screening, we chose to restrict our analysis to populations without chronic disease to assess trial design elements relevant to the general population. This, however, did not preclude those with metabolic syndrome for whom obesity is a common component. Secondly, the systematic review we assessed also contained studies that was excluded based on our stringent selection criteria. Given the large volume of studies omitted based on our pragmatic exclusion criteria, we cannot generalize whether the same variations in study design could be applied to other studies of more specific diseases or genders. Thirdly, we recognize that delivery modalities alter the pharmacokinetics of resveratrol, resulting in targeted effects on different tissue types. As such, variable design strategy may be relevant to drug development, and subject to different regulatory requirements as directed by regulatory agencies. Finally, we are cautious to not overstate our findings given the small number of studies that were available for assessment. We suggest that future studies involving meta-analysis should utilize ClinicalTrials.gov or other appropriate RCT registries to assess whether specific trial design elements are appropriate for inclusion into their analysis. Additionally, a meta-analysis should carefully consider trial design to better select relevant studies and reduce heterogeneity.

Systematic reviews inherently suffer from data heterogeneity, which in some cases may be attributed to a poor trial design. Therefore, the general indication of effectiveness by meta-analyses is not sufficient if trial designs have not formed part of the inclusion and exclusion criteria. Greater stringency in selecting appropriate studies in terms of their design is an easy and fitting first step in avoiding such potential serious error in conducting a flawed systematic review that contributes to understanding whether there is enough power across pooled studies to yield an overall positive effect for a treatment. The use of ClinicalTrials.gov is one such proposed approach but a better definition of inclusion and exclusion criteria for all RCTs deposited is necessary. This information should then be incorporated to refine the studies included, thereby leading to improved outcomes from cross-study analyses. We further recommend, for all future RCT designs, the inclusion of standardized methodology and data collection regarding inclusion and exclusion criteria, primary and secondary endpoints, sample size and power statistics, therapeutic intervention (dosage, duration, mode of delivery). Registries such as ClinicalTrials.gov ideally serve as a primary resource to assess clinical trial designs, particularly if they are non-published trial studies or protocols.

## 5. Conclusions

In conclusion, heterogeneity in trial design using resveratrol as an exemplar therapeutic intervention suggests that weight-loss-related outcomes are difficult to interpret and cross-validate. Indeed, conclusions drawn from human studies have been inconsistent, which may in part be attributable to the heterogeneity of study design including major differences in sample population, age, sex, BMI, underlying health conditions, resveratrol administration and end-point measures. Inconsistencies in study design pose a challenge for systematic reviews and meta-analysis studies to accurately assess effect size and identify the most appropriate interventional strategies for the clinical use of resveratrol on obesity in the general population. We propose the need to establish standardized guidelines for depositing relevant information in future RCTs, and best practices for selecting studies to include in subsequent analyses based on trial design elements.

## Figures and Tables

**Table 1:** Summary of clinical trial characteristics.

NCT Number	*n* (Study Population Size)	Inclusion Criteria				Exclusion Criteria	Resveratrol		Weight Loss Measure			Biomarkers Tested (Related to Weight Loss)
		Gender	Age Range (Years)	BMI (kg/m^2^)	Population	Population	Resveratrol Dosage (per Day)	Interval	Outcome Measures	Type of Weight Loss Measures	Timing/Interval of Measures	
NCT01412645	76	Male	30–60	Not Specified	MetS	T2DM/Chronic Condition/Malignancy	Resveratrol 1000 mg/Resveratrol 150 mg	2 times/day for 120 days	**Other:** Body composition/Biomarkers	DXA/MR	16 weeks	**General:** Inflammatory Biomarkers/Fat- and sugar-metabolism/Bone-metabolism.
NCT01446276	26	Male	25–65	>28	NAFLD	Malignancy	Resveratrol 1500 mg	3 times/day for 180 days	**Secondary:** Body composition **Other:** Lipid turnover/Liver fat content/Lipase activity and fat cell size	DXA/MR	24 weeks	**Specific:** Hepatic VLDL-TG secretion and peripheral VLDL-TG clearance/Basal and insulin stimulated free fatty acid and glucose turnover/VLDL-TG oxidation
NCT01717820	40	Female	18-55	Not Specified	PMW		Whole grape powder 46 g	2 times/day for 84 days	**Primary:** Body composition **Secondary:** Adipose metabolism	Anthropometric measurements/DXA	12 weeks	
NCT02633150	23	Female & Male	50–65	>30	PMW	T2DM/Chronic Condition	Supplementation with red grapes polyphenol (Volume not specified)	56 days	**Others:** Body composition/Biomarkers	Anthropometric measurements/DXA	8 weeks	**Specific:** Adipokines sensitivity/Clamp hyperinsulinemic-euglycemic/Lipopolysaccharides/Mitochondrial activity or cell characterization (adipocyte precursor and muscle)
NCT02114892	24	Female & Male	30–50	<39.9	MetS	Chronic Condition/Pregnancy	Resveratrol 1500 mg	3 times/day for 90 days	**Primary:** Waist circumference/Biomarkers **Secondary:** Weight/BMI/Biomarkers	Anthropometric measurements	12 weeks	**Specific:** Triglycerides levels/High density lipoprotein levels/Low density lipoproteins
NCT03062163	66	Female & Male	Not Specified	>23			Dermal Patch loaded with resveratrol (Volume not specified)	14 days	**Primary:** Fat thickness	Ultrasound	2 weeks	
NCT02565979	42	Female & Male	50–70/ 40–70	27–35	PMW	T2DM/Hypertension/Chronic Condition	Resveratrol 150 mg	2 times/day for 180 days	**Primary:** Body composition/Biomarkers	DXA	24 weeks	**General:** Glucose Tolerance/Intra-hepatic lipid content/Blood plasma markers

MetS, metabolic syndrome; NAFLD, non-alcoholic fatty liver disease; PMW, post-menopausal women; T2DM, type 2 diabetes mellitus; DXA, dual-energy X-ray absorptiometry; MR (includes MRI and MRS), Magnetic Resonance Imaging and Magnetic Resonance Spectroscopy.

## Data Availability

Data in this study will be made available upon reasonable request to the corresponding author (C.S.M.).

## Supplementary Materials

The following supporting information can be downloaded at: https://www.mdpi.com/article/10.3390/nu14071424/s1, Table S1. Comparison of trial design elements in studies registered on clinicaltrials.gov to those included in the most recently published meta-analysis.
